# Environmental risk factors for chronic kidney disease of non-traditional causes in tropical coastal areas: A systematic review and meta-analysis

**DOI:** 10.1371/journal.pntd.0013056

**Published:** 2025-05-06

**Authors:** Hsiao-Yu Yang, Kai-Chieh Wen, Ping-Fang Chiu, Wan-Chin Chen, Teng-Hsiang Chang, Che-Jui Chang, Wei-Hung Hsu, Shin-Chien Chen

**Affiliations:** 1 Institute of Environmental and Occupational Health Sciences, National Taiwan University, Taipei, Taiwan; 2 Department of Public Health, National Taiwan University College of Public Health, Taipei, Taiwan; 3 Population Health Research Center, National Taiwan University, Taipei, Taiwan; 4 Department of Family Medicine, National Taiwan University Hospital Yunlin Branch, Yunlin, Taiwan; 5 Department of Environmental and Occupational Medicine, National Taiwan University Hospital, Taipei, Taiwan; 6 Division of Nephrology, Department of Internal Medicine, Changhua Christian Hospital, Changhua, Taiwan; 7 Department of Post Baccalaureate, College of Medicine, National Chung Hsing University, Taichung, Taiwan; 8 Department of Family Medicine, Changhua Christian Hospital, Changhua, Taiwan; 9 Department of Family Medicine, National Taiwan University Hospital Hsin-Chu Branch, Hsinchu, Taiwan; 10 Department of Family Medicine, College of Medicine, National Taiwan University, Taipei, Taiwan; 11 Department of Family Medicine, Hsinchu Mackay Memorial Hospital, Hsinchu, Taiwan; 12 Department of Occupational Medicine, Min-Sheng General Hospital, Taoyuan, Taiwan; University of Massachusetts Amherst, UNITED STATES OF AMERICA

## Abstract

**Background:**

Chronic kidney disease of non-traditional etiology (CKDnt) is a neglected tropical disease prevalent in tropical coastal areas. First reported in the 1990s along the Pacific coast of Central America, its spread to other regions has raised concerns about environmental risk factors, particularly heat stress. However, the relationship between elevated ambient temperatures and CKDnt remains uncertain. The study aimed to identify risk factors for chronic kidney disease (CKD) in regions affected by the CKDnt epidemic and to investigate the relationship between ambient temperatures and CKD risk.

**Methods:**

We conducted a systematic review and meta-regression of CKD in agricultural regions where CKDnt is endemic, covering studies published between January 2010 and October 2023, followed by a meta-analysis to estimate the effect of traditional and non-traditional risk factors for CKD. A meta-regression was used to examine the relationship between geological latitude and ambient temperature on CKD.

**Results:**

We screened 1,327 articles, with 28 articles meeting the inclusion criteria. The pooled OR for CKD in the agricultural population compared to the non-agricultural population was 2.12 (95% CI 1.75‒2.58, I2 = 85.1%). Significant non-traditional kidney disease risk factors for CKD included drinking well water (OR = 2.75, 95% CI 2.04‒3.70), malaria (OR = 2.64, 95% CI 1.44‒4.83), low water intake (pooled OR = 2.06, 95% CI 1.17‒3.63), water sources (pooled OR = 1.50, 95% CI 1.11‒2.02), agrochemicals (OR = 1.50, 95% CI 1.26‒1.77), heat exposure (OR = 1.46, 95% CI 1.37‒1.55), alcohol consumption (OR = 1.27, 95% CI 1.11‒1.46), and low BMI. The meta-regression indicates that geographic latitude and temperature are statistically significant moderators of CKD risk, with a higher risk observed in studies conducted at lower latitudes closer to the equator (QM-test = 10.11, df = 1, P < 0.05). Temperature is a significant moderator (QM-test = 44.36, df = 1, P = 0.04) with 1°C increase in the CKDnt epidemic region associated with an 8% increase in CKD risk (OR = 1.08, 95% CI 1.01–1.16).

**Conclusion:**

CKDnt is a multifactorial tropical disease driven by heat exposure, infectious diseases, physically demanding work without adequate hydration, water contamination, and agrochemical exposure. Addressing these factors is essential for developing effective occupational health policies and tailored prevention programs to reduce CKDnt among high-risk agricultural populations in tropical endemic regions.

## Summary

Chronic Kidney Disease of Non-Traditional Etiology is a Neglected Tropical Disease Influenced by Geographic Latitude and Temperature

## Introduction

Chronic kidney disease (CKD) is a prevalent and growing global health concern. The global prevalence of chronic kidney failure is estimated to be 11.1% and the global number of individuals with CKD is 843.6 million [1]. The increase in numbers is partly due to factors such as aging and rising rates of diabetes and hypertension, but the relationship between global warming and CKD is beginning to be emphasized [2].

Chronic kidney disease of non-traditional etiology (CKDnt), also known as chronic kidney disease of unknown etiology (CKDu), is a significant health concern affecting agricultural communities in tropical climates worldwide. Initially termed Mesoamerican nephropathy (MeN) due to its emergence in Central American countries like El Salvador and Nicaragua [3,4], where heat stress was suspected as a primary cause, the disease has also been reported in tropical regions such as Sri Lanka’s North Central Province, with prevalence rates ranging from 6% to 15%, primarily among paddy farmers [5,6]. In Taiwan, the prevalence of CKDnt was 1.5% in farmers and 0.4% in nonfarmers [7]. Non-traditional risk factors may contribute to the varying prevalence of CKDnt. These include occupational hazards such as prolonged exposure to heat stress, dehydration, and agrochemicals during agricultural work; environmental factors like potential exposure to heavy metals and toxins in drinking water; and socioeconomic factors, including limited access to healthcare and poor working conditions. The exact etiology remains unclear, necessitating further research to identify specific causes and develop effective prevention and treatment strategies.

In the 1990s, an epidemiologic study in El Salvador reported that young residents living near coastal areas, many of whom were agricultural workers, were at high risk of developing kidney diseases [8]. For more than a decade, epidemics of CKD have been observed in many Central American countries [9–14], and have been reported in Asian countries [15–17]. Although heat stress due to global warming is suspected to be associated with a new epidemic of kidney disease in this tropical coastal region, no epidemiologic study has compared the effect of ambient temperature change on the risk of kidney disease. A clear association between elevated temperatures and CKDnt has yet to be established. As CKDnt is mostly reported in agricultural communities, pesticides [18], heavy metal contamination [19], and betel nut chewing are also suspected to be the causes of CKDnt in rural communities [17]. The impact of traditional risk factors (e.g., diabetes, hypertension, pesticides, heavy metals, and pesticides) and non-traditional risk factors (e.g., elevated temperature) for CKDnt in tropical coastal areas remain unclear.

The study aimed to identify risk factors for CKD in regions affected by the CKDnt epidemic and to investigate the relationship between ambient temperatures and CKD risk.

## Methods

### Study design and setting

We conducted a systematic review, meta-analysis, and meta-regression of heat stress-related chronic kidney diseases. We included epidemiological studies that analyzed CKD or CKDnt in adult or adolescent agricultural workers or populations from agricultural communities. We formulated our research questions using the PECO structure [20]: (1) Population—adult or adolescent human subjects, including agricultural workers, or in populations sampled from agricultural communities. (2) Exposure/risk factors—exposure to occupational factors (i.e., agrochemicals), environmental factors (i.e., water source), and personal factors (i.e., diabetes, hypertension, gender, alcohol, smoking, BMI, family history of kidney disease, and use of NSAIDs). (3) Comparison—subjects with/without exposure. (4) Outcomes—CKD and CKDnt. We followed the PRISMA 2020 guidelines to report our review process and findings (Table A in S1 Text).

### Eligibility criteria

*Inclusion Criteria:*

1. *Study Design:* We included observational studies, specifically cross-sectional, case-control, and cohort studies, as these designs provide valuable epidemiological evidence on the association between agricultural work and CKD/CKDnt. These studies were published between January 2010 and October 2023.
2. *Study Population:* Studies must have examined agricultural workers or individuals from agricultural communities. This criterion ensures that the findings are directly applicable to populations at risk due to agricultural exposures.
3. *Outcome Measures:* The primary outcomes of interest were CKD and CKDnt. Kidney function was assessed using the estimated glomerular filtration rate (eGFR) < 60 mL/min/1.7m^2^, and diagnoses were determined according to the Kidney Disease Improving Global Outcomes (KDIGO) guidelines to maintain consistency and comparability across studies.

*Exclusion Criteria:*

1. *Non-Human and In Vitro Studies:* Studies conducted solely on animals or *in vitro* models were excluded, as they do not provide direct evidence relevant to human populations.
2. *Lack of Outcome Definition or Analysis:* Studies that did not provide a detailed description of CKD or CKDnt outcomes, or those that did not analyze kidney disease according to the KDIGO guidelines, were excluded to ensure consistency in disease classification.
3. *Acute decline in kidney function:* Studies that focused on acute decline in kidney function were excluded, as our research aimed to assess chronic kidney disease.
4. *Case Reports and Articles Without Statistical Analyses:* Case reports lacking statistical analyses were excluded, as they do not provide population-based evidence. Similarly, letters, review articles, conference abstracts, editorials, and commentaries were not included because they do not present original research findings.
5. *Insufficient Data for Meta-Analysis:* Studies that lacked sufficient information to construct a 2 × 2 contingency table (e.g., missing exposure or outcome data) were excluded to ensure statistical robustness in meta-analysis. Additionally, studies that assessed kidney function using mean differences as the effect measure were excluded, as they are not suitable for pooling with studies that use categorical outcome measures.
6. *Lack of a Measure of Association:* Studies that did not report a measure of association (e.g., odds ratio, relative risk, or hazard ratio with a 95% confidence interval) between agricultural work and CKD/CKDnt were excluded, as they do not provide quantitative evidence for risk assessment.
7. *Inappropriate comparison*: Studies in which the effect size was derived from comparisons of high versus low altitude, WBGT zones, specific regions, or specific conditions (e.g., greenhouse vs. field workers); those reporting odds ratios for specific heat index increases; or those using eGFR < 90 ml/min/1.73m² as an outcome, making comparisons with other studies infeasible.
8. *Studies not assessing heat exposure risk: Given that this study aims to assess the association between elevated temperature, occupations, and environmental risk factors for CKD and CKDnt, the inclusion of studies focusing on these specific exposures is essential. Studies that did not assess the risk associated with farming or heat-exposure occupations were excluded*.

### Information sources

Given the numerous challenges in obtaining standardized CKDnt prevalence estimates in low- and middle-income countries, including the asymptomatic nature of CKDu in early stages, variability in awareness and access to renal care, limitations of routine screening tools, and inconsistencies in GFR estimation methods [6], we limited our inclusion to studies published after 2010 to enhance reliability. By restricting our search to studies published after 2010, we aimed to include research conducted with more standardized diagnostic criteria and improved methodologies, ensuring greater comparability across studies. The bibliographic databases used for this study were PubMed, Embase, and Web of Science. We searched the articles published between January 2000 and February 2025. The results of literature searches were stored in EndNote X9 and checked for duplicates.

### Search strategy

The search terms were divided into two categories. The first category included studies with population-related text words, such as “agricultural worker”, “farmer” and “sugarcane cutter”. The second included studies with words related to the decline in kidney function, such as “kidney disease”, “kidney injury”, “kidney failure”, “CKD of unknown etiology (CKDu)”, “CINAC (chronic interstitial nephritis of agricultural communities)”, “Mesoamerican Nephropathy”, “Uddanam Nephropathy”, “Sri Lankan Nephropathy”, etc. We further examined studies that cited any of our initially selected studies and incorporated the names of countries that have reported CKDu. The full details of the search strategy are characterized in Table B in S1 Text.

### Selection process

Six reviewers (K.-C. W., T.-H. C., C.-J. C., W.-C. C., W.-H. H., S.-C. C.) independently reviewed the study titles and abstracts and investigated the discrepancies until a consensus was reached by discussion with the senior nephrologist P.-F. C. and the corresponding author (H.-Y. Y.). We also searched for studies that cited any of the initially included studies and their references. However, none of these additional articles met the inclusion criteria. For each article, we contacted the corresponding author if further information was needed. Those studies that did not receive a response were excluded from our review.

### Data collection process

Six reviewers (K.-C. W., T.-H. C., C.-J. C., W.-C. C., W.-H. H., S.-C. C.) independently extracted the following characteristics from each included study: study title, first author’s name, year of publication, article type, country, region, number of participants, demographics of the participants, risk exposures, study protocol, and outcome measures. Inconsistencies were then investigated until a consensus was reached through discussion with the corresponding author (H.-Y. Y.). Finally, the details of the completed data elements were summarized in Table C in S1 Text.

### Data items

We collected data items of data on the country where the study was conducted, sampling method, age distribution, number of male and female subjects, outcome measure, effect size and 95% confidence interval, predictor, and adjusted variables. The definition of CKD follows the KDIGO guidelines as the presence of any of the following for > 3 months: (1) Markers of kidney damage (one or more): albuminuria (albumin excretion rate ≥30 mg/24 hours or albumin to creatinine ratio ≥ 30 mg/g [≥ 3 mg/mmol]), urine sediment abnormalities, electrolyte and other abnormalities due to tubular disorders, abnormalities detected by histology, structural abnormalities detected by imaging, history of kidney transplantation. (2) Decreased glomerular filtration rate: glomerular filtration rate (eGFR) < 60 mL/min/1.73m^2^” [21]. Since most cross-sectional studies did not obtain repeated eGFR measurements at intervals of at least three months, we defined CKD based on a single eGFR measurement of < 60 mL/min/1.73 m², irrespective of chronicity. CKDnt was defined as CKD without diabetes mellitus, hypertension, or glomerulonephritis [22].

We collected temperature data from the reviewed articles to explore the association between CKD and elevated temperatures. For studies that did not report temperature, we estimated the mean temperature using data from the nearest climate station corresponding to the study period. For studies spanning multiple years, we used the average annual temperature provided by the National Oceanic and Atmospheric Administration (NOAA) [23]. Additionally, we recorded the latitude of the study area for meta-regression analysis. Since most studies did not provide temperature data, we also obtained the latitude of the study locations as a proxy indicator for temperature in our analysis. For studies conducted in multiple locations, we used the average temperature and latitude of those locations.

### Risk of information bias assessment

Data on CKD risk factors, including diabetes, hypertension, agrochemical exposure, heavy metals, and heat stress, were extracted from the studies. Some studies relied on direct measurements, while others used questionnaires, and some did not specify their data sources. Based on the reliability of the information, we categorized the risk of information bias into three levels: low, moderate, and high. A “low risk” classification indicates that the information was obtained through objective measurements, whereas an “unclear risk” suggests reliance on questionnaires or self-reports. A “high risk” designation signifies that the study did not mention these variables.

### Effect measures

We obtained odds ratios (ORs) or relative risks (RRs) for CKD among agricultural workers or farmers exposed to heat. Additionally, we collected ORs or RRs for both traditional risk factors—such as diabetes, hypertension, NSAID use, a family history of kidney disease, male gender, and smoking—and non-traditional risk factors, including heat exposure, agrochemicals, alcohol consumption, Betel chewing, drinking well water, malaria, low water intake, water sources, high physical demands, and BMI.

### Synthesis methods and statistical analysis

OR and RRs were pooled together for the meta-analysis. We used Cochrane’s Q-test and I-square test to assess heterogeneity. The mean OR was estimated using a random-effects model when statistically significant heterogeneity was present and a common-effects model when it was absent. Publication bias was evaluated by Egger’s linear regression using the funnel plot. To assess whether temperature or latitude modifies the association with CKD, we conducted a meta-regression analysis to examine the relationship between the OR of CKD and both temperature and latitude. The variance between studies was estimated using restricted maximum likelihood and the proportion of variance explained by the meta-regression model was estimated using the R^2^ statistic. An omnibus test (Q_M_-test) was used to examine whether temperature or latitude is a significant moderator of CKD. We conducted a meta-regression analysis using a mixed-effects model [24] to determine whether temperature and latitude act as effect modifiers contributing to the heterogeneity in risk estimates across studies. A corresponding diagram is provided to visually illustrate this relationship [24]. The meta-analysis procedure of our research uses “meta”, “metafor”, and “metagen” in R 4.2.1 software. In this study, we set the significance level at 5%. We have included confidence intervals for all statistical measures to assess certainty.

### Sensitivity analysis

We conducted a stratified analysis of the OR for chronic kidney disease by country and region. Additionally, we performed a sensitivity analysis focusing exclusively on CKDnt to assess the pooled OR by country and region.

This study was approved by the Research Ethics Committee (No. 202004HM030) and patient consent was not required.

## Results

**Fig 1:**
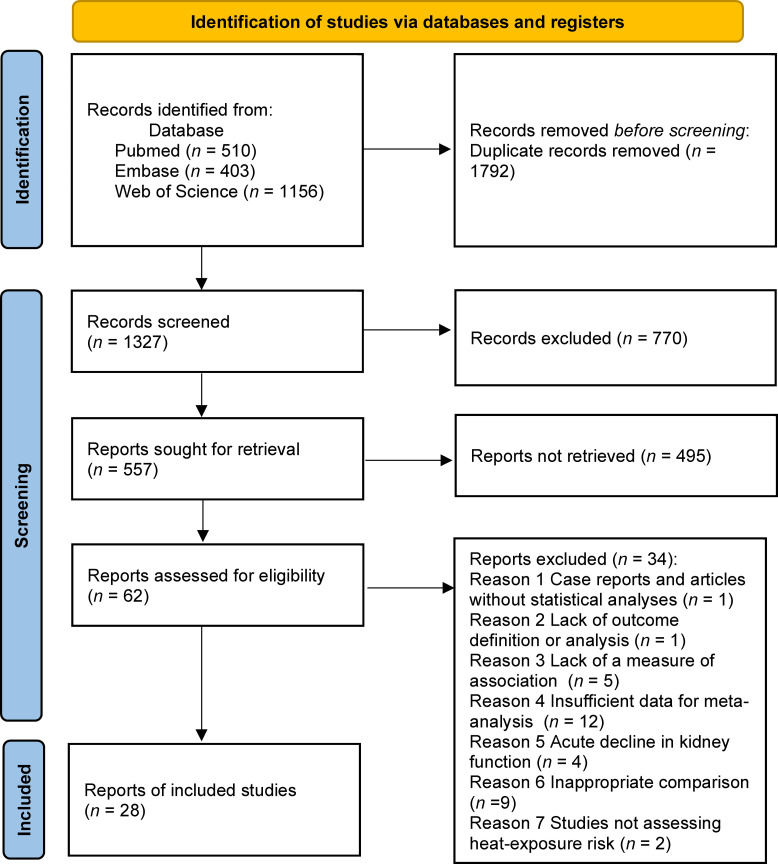
PRISMA 2020 flow diagram. Legend: This is the plot of the overall steps of our study, which follows the PRISMA 2020 flow diagram.

There were 1,327 relevant articles published between January 2000 and February 2025. After reviewing the article’s title and abstract of the articles, we identified 557 studies on CKD in the agricultural population. Based on our exclusion criteria, we included 28 studies spanning seven tropical countries (Fig 1). While most of these countries are entirely tropical, Taiwan, as well as parts of Mexico and India, have subtropical regions. The mean temperature of in the affected areas ranged from 21.62°C to 31.7°C, while the latitude ranged from 7.29° to 23.8°. The characteristics of the included study are summarized in Table 1. Most of the cross-sectional studies were community-based, recruited participants from different age groups, and investigated a variety of exposures. Data on the country of study, sampling method, age distribution, number of male and female subjects, predictor, and adjusted variables of the included studies are summarized in Table C in S1 Text. Most studies were community surveys without random sampling. Most studies used a single blood test and eGFR < 60mg/dL as the predictor (Table C in S1 Text).

**Table 1:** Summary characteristics of included studies eligible for the meta-analysis.

Study	Country	Region	Latitude	Temperature	Exposure	Study type	Outcome	Effect Size	LCI	UCI
1. Sanoff et al. 2010 [25]	Nicaragua	Central America	12.1	25.88	agricultural fieldwork	cross-sectional	CKD	2.48	1.59	3.89
2. Torres, et al. 2010 [9]	Nicaragua	Central America	12.1	25.88	banana/sugarcane village	cross-sectional	CKD	3.39 (M)	1.67 (M)	6.91 (M)
								2.42 (F)	0.79 (F)	7.44 (F)
3. Athuraliya^,^ et al. 2011 [26]	Sri Lanka	South Asia	8	27.25	farmer	cross-sectional	CKD	2.60	1.90	3.40
4. O’Donnell, et al. 2011 [10]	Nicaragua	Central America	12.51	25.88	agricultural work	case-control	CKD	1.00	0.44	2.27
5. Orantes, et al. 2011 [11]	El Salvador	Central America	14	25.23	agricultural worker	cross-sectional	CKD	1.35	0.63	2.88
6. Peraza, et al. 2012 [12]	El Salvador	Central America	14	25.23	working in coastal sugarcane or cotton	cross-sectional	CKD	3.10 (M)	2.00 (M)	5.00 (M)
								2.30 (F)	1.40 (F)	3.70 (F)
7. Nanayakkara, et al. 2014 [16]	Sri Lanka	South Asia	8.54	27.25	farming	case-control	CKDnt	9.17	4.18	20.00
8. Orantes, et al. 2014 [27]	El Salvador	Central America	14	25.23	agricultural worker	cross-sectional	CKD	2.30	1.85	2.85
9. Raines, et al. 2014 [28]	Nicaragua	Central America	12.37	25.88	>365 lifetime days harvesting any crop	cross-sectional	CKD	2.29	0.85	6.20
10. Vela, et al. 2014 [29]	El Salvador	Central America	13.58	25.23	agricultural worker	cross-sectional	CKD	0.96	0.52	1.77
11. Lebov, et al. 2015 [30]	Nicaragua	Central America	12.25	25.88	agricultural worker	cross-sectional	CKD	3.99	2.2	7.24
12. Jayasumana et al. 2015 [31]	Sri Lanka	South Asia	8.6	28.5	farming	case-control	CKDnt	3.12	1.74	5.61
13. Siriwardhana, et al. 2015 [32]	Sri Lanka	South Asia	8	28.07	working >6 h in the field under sun	cross-sectional	CKDnt	8.56	2.27	11.41
14. Anand, et al 2019 [33]	Sri Lanka	South Asia	7.29	27.25	farming	longitudinal	CKDnt	2.00	1.20	2.90
15. Orantes-Navarro, et al. 2019 [34]	El Salvador	Central America	14	25.23	farmer	cross-sectional	CKDnt	2.62	1.70	4.04
16. Herrera-Valdés, et al. 2019 [35]	El Salvador	Central America	13.3	25.21	farming	cross-sectional	CKD	2.0	1.5	2.5
17. Ruwanpathirana, et al. 2019 [6]	Sri Lanka	South Asia	8.53	26.7	farming	cross-sectional	CKDnt	2.1 (M)	0.6 (M)	7.8 (M)
							CKDnt	1.5 (F)	0.7 (F)	3.3 (F)
18. Tatapudi, et al. 2019 [36]	India	South Asia	18.87	27.9	farmer	cross-sectional	CKDnt	1.23	0.76	1.99
19. Ferguson, et al. 2020 [37]	Nicaragua	Central America	11.33	25.88	worked in agriculture	longitudinal	CKD	1.53	0.76	3.09
20. Gummidi, et al. 2020 [38]	India	South Asia	18.3	23.65	outdoor worker	cross-sectional	CKD	1.12	0.90	1.39
21. Aguilar-Ramirez, et al. 2021 [39]	Mexico	Central America	19.12	31.7	agriculture	cross-sectional	CKD	4.38	1.05	18.20
22. Chang, et al. 2021 [40]	Taiwan	East Asia	23.8	21.62	farmer	cross-sectional	CKDnt	1.45	1.10	1.90
23. Miller, et al. 2021 [41]	Guatemala	Central America	14.46	23.65	sugarcane farmer	cross-sectional	CKD	0.57	0.06	5.17
24. Chang, et al. 2023 [15]	Taiwan	East Asia	23.5	21.62	farmer	case-control	CKDnt	1.09	0.98	1.22
25. Figueroa-Solis, et al. 2023 [42]	Guatemala, Nicaragua	Central America	12.43	23.9	exposure to heat	cross-sectional	CKDnt	1.37	0.57	3.3
26. Sinha,et al. 2023 [43]	India	South Asia	23.23	26.7	uncomfortable heat exposure	cross-sectional	CKD	2.52	0.67	9.54
27. Strasma, et al. 2023 [44]	Nicaragua	Central America	12.43	29.17	current agricultural occupation	cross-sectional	CKDnt	5.72	2.34	13.99
28. Gonzalez-Quiroz, et al. 2024 [45]	Nicaragua	Central America	12.43	27.8	sugarcane farmers	cohort study	CKD	1.76	1.09	2.82

**Fig 2:**
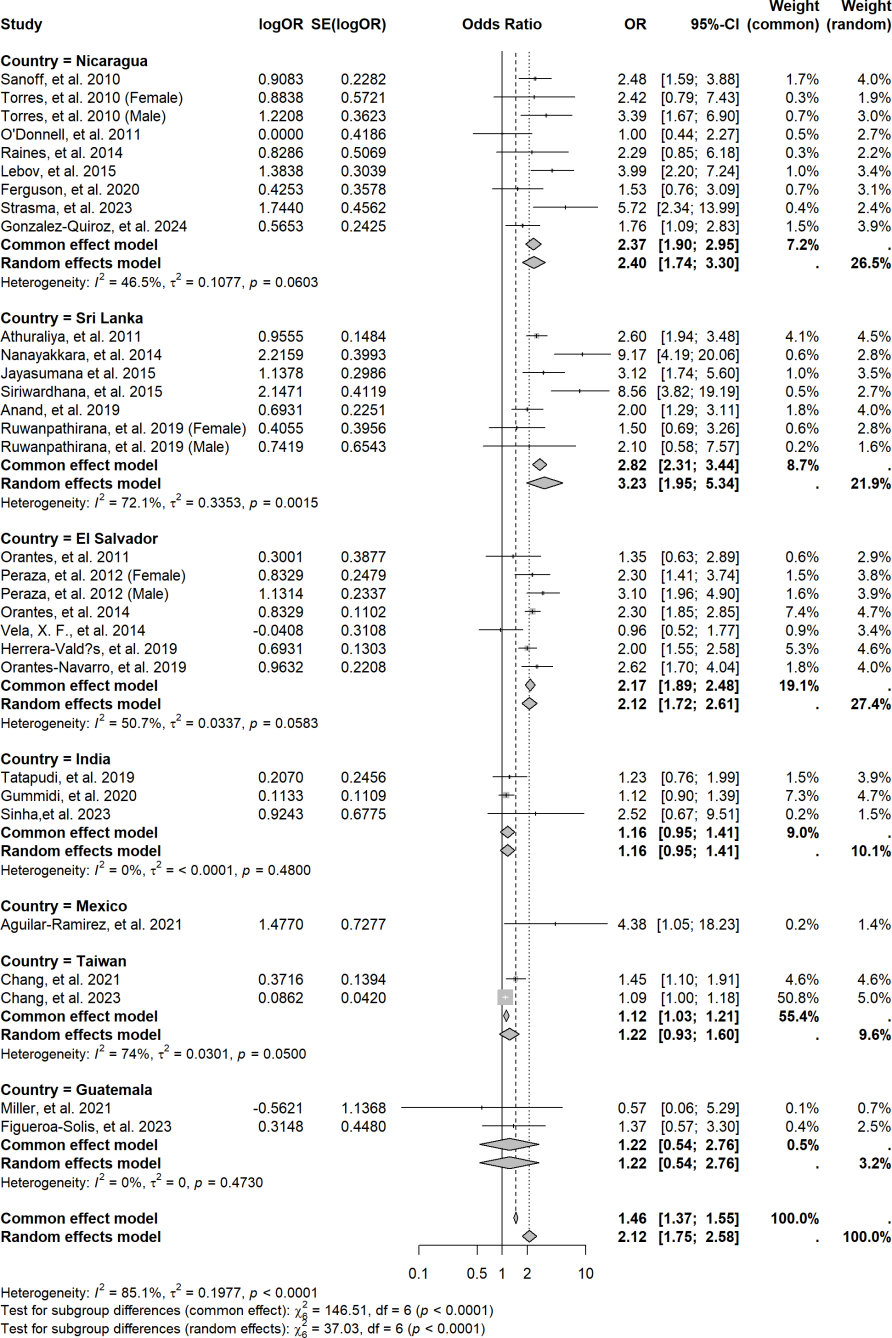
Forest plot of pooled odds ratio for CKD in countries experiencing CKDnt epidemics, stratified by country. Legend: Forest plot showing pooled odds ratios (OR) for chronic kidney disease (CKD) across countries experiencing CKD of non-traditional causes (CKDnt). Each study’s log odds ratio (logOR) and standard error (SE) are plotted, with subgroup analyses by country. Common and random-effects models are presented, with heterogeneity statistics (I²) for each subgroup. The diamonds indicate pooled estimates, with the center representing the OR and the width denoting the 95% confidence interval.

Fig 2 presents a pooled ORs or RRs for CKD among agricultural workers or farmers exposed to heat in countries where CKDnt is endemic. The pooled OR for CKD in the agricultural population compared to the non-agricultural population was 2.12 (95% CI 1.75‒2.58, *I*^*2*^ = 85.1%). The funnel plot indicates a slight publication bias (Fig A in S1 Text). Except for one small study in Guatemala, which reported an unusually low risk of CKD [41], smaller studies tended to report a higher risk, leading to a slightly asymmetric scatter plot.

### Regional differences

**Fig 3:**
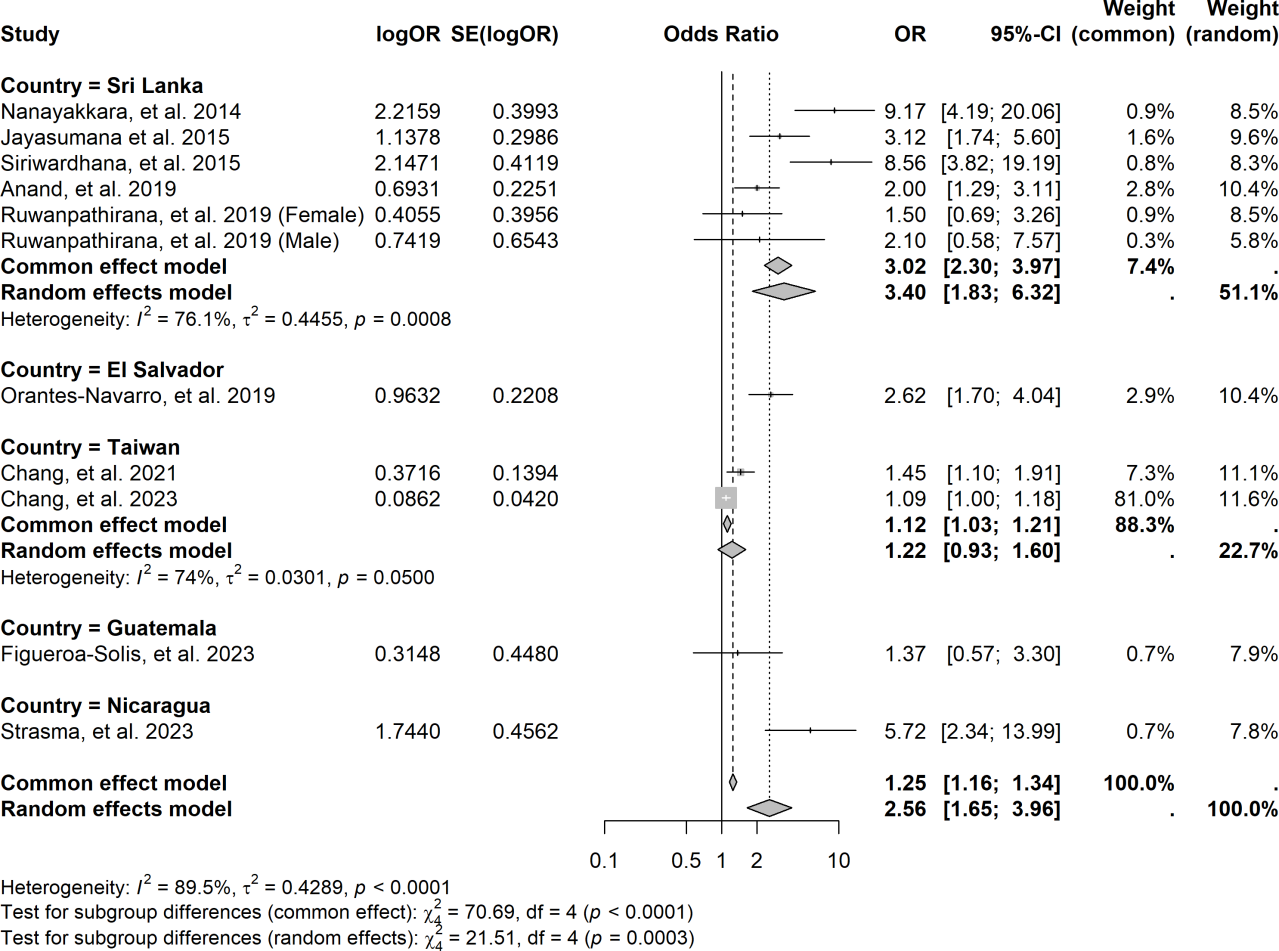
Forest plot of pooled odds ratio for chronic kidney disease of CKDnt, stratified by country. Legend: Forest plot of pooled odds ratios (OR) for CKDnt, showing individual study estimates, 95% confidence intervals, and common/random effects models.

The sensitivity analysis, focusing exclusively on CKDnt, assessed the pooled OR by country shows that the pooled OR for CKDnt was 2.56 (95% CI 1.65‒3.96) and Sri Lanka had the highest pooled OR for CKDnt (random effect OR = 3.40, 95% CI 1.83‒6.32, *I*^*2*^ = 89.5%) followed by El Salvador (OR = 2.62, 95% CI 1.70‒4.04) (Fig 3). When stratified by region, South Asia had the highest pooled OR for CKD (random effects OR = 2.49, 95% CI 1.58‒3.95, *I*^*2*^ = 85.5%), followed by Central America (random effects OR = 2.19, 95% CI 1.84‒2.60, *I*^*2*^ = 42.0%), and East Asia (common effect OR = 1.12, 95% CI 1.03‒1.21, *I*^*2*^ = 74.0%). Additionally, we performed a sensitivity analysis focusing exclusively on CKDnt to assess the pooled OR by region. South Asia had the highest risk for CKDnt (random effect OR = 3.40, 95% CI 1.83‒6.32, *I*^*2*^ = 76.1%), followed by Central America (common effect OR = 2.67, 95% CI 1.35‒5.43, *I*^*2*^ = 60.1%).

### Non-traditional and traditional risk factors

**Fig 4:**
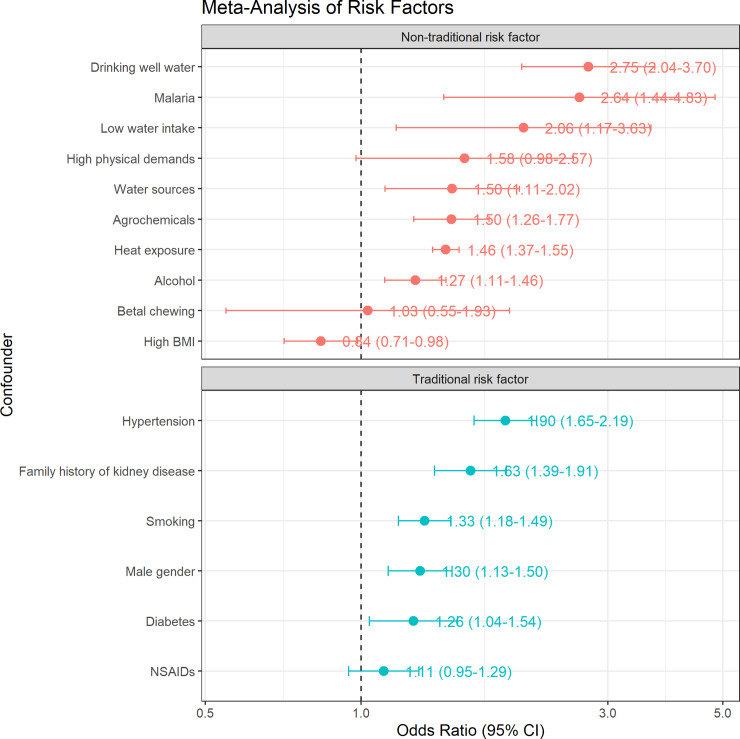
The pooled odds ratio of non-traditional and traditional risk factors for CKD in epidemic regions. Legend: Pooled odds ratios of CKD risk factors, categorized into non-traditional and traditional factors, with 95% confidence intervals.

Fig 4 shows the effect of CKD risk factors reported in the CKDnt epidemic areas. Significant non-traditional kidney disease risk factors for CKD included drinking well water (pooled OR = 2.75, 95% CI 2.04‒3.70), malaria (pooled OR = 2.64, 95% CI 1.44‒4.83), low water intake (pooled OR = 2.06, 95% CI 1.17‒3.63), water sources (pooled OR = 1.50, 95% CI 1.11‒2.02), agrochemicals (pooled OR = 1.50, 95% CI 1.26‒1.77), heat exposure (pooled OR = 1.46, 95% CI 1.37‒1.55), alcohol consumption (pooled OR = 1.27, 95% CI 1.11‒1.46), and low BMI. Significant traditional kidney disease risk factors for CKD were hypertension (pooled OR = 1.90, 95% CI 1.65‒2.19), family history of kidney disease (pooled OR = 1.63, 95% CI 1.39‒1.91), smoking (pooled OR = 1.33, 95% CI 1.18‒1.49), male gender (pooled OR = 1.30, 95% CI 1.13‒1.50), and diabetes (pooled OR = 1.26, 95% CI 1.04‒1.54).

### Environmental factors

**Fig 5:**
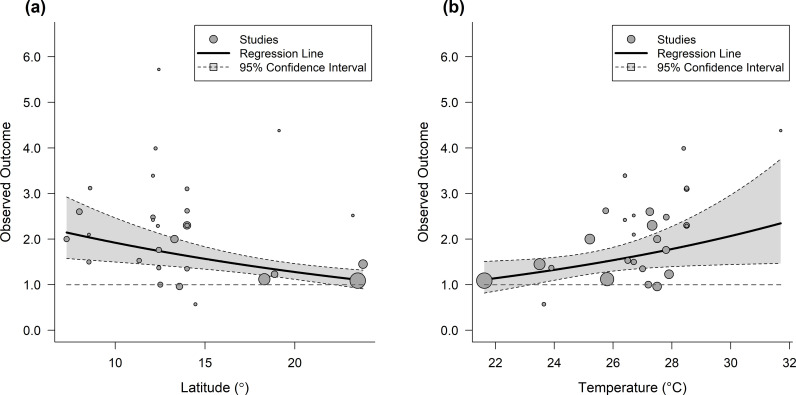
Meta-regression plots showing the association between (a) latitude and (b) temperature with observed outcomes. Legend: Each circle represents a study, with size proportional to weight. Regression lines and 95% confidence intervals are shown.

The meta-regression indicates that geographic latitude and temperature are statistically significant moderators of CKD risk (Fig 5), with a higher risk observed in studies conducted at lower latitudes closer to the equator (QM-test = 10.11, df = 1, P < 0.05) (Table D in S1 Text). Elevated temperature is a significant moderator (QM-test = 44.36, df = 1, P = 0.04) with 1°C increase in the CKDnt epidemic region associated with an 8% increase in CKD risk (OR = 1.08, 95% CI 1.01–1.16) (Table E in S1 Text).

### Risk of information bias

The risk of bias assessment reveals significant gaps in the literature regarding key health risk factors. Notably, heat stress was frequently unmentioned or inadequately assessed, with most studies showing a high risk of bias, highlighting the need for direct temperature monitoring and physiological heat stress evaluations. Similarly, agrochemical exposure lacked objective assessments, relying largely on self-reported questionnaires, which introduces potential misclassification and recall bias. While diabetes and hypertension were more consistently reported, the methods of assessment varied, with some studies using objective medical diagnoses and others relying on self-reported data, underscoring the necessity for standardized clinical measurements. Heavy metal exposure was also largely absent from direct assessments, emphasizing the importance of environmental and biomonitoring methods (Fig B in S1 Text).

## Discussion

Although CKDnt is not widely recognized as a neglected tropical disease (NTD), it is highly prevalent in hot, humid, rural agricultural communities. Our meta-analysis and meta-regression provide evidence that CKD risk in epidemic regions increases with higher temperatures and lower latitudes, further supporting the classification of CKDnt as an NTD. By synthesizing evidence of non-traditional risk factors primarily affecting underserved communities in tropical agricultural areas, recognizing non-traditional risk factors for CKDnt could help allocate necessary resources for prevention, mitigation, and worker protection in high-risk environments.

Comparing our findings with existing meta-analysis for CKDnt, Our finding aligns with a meta-analysis by the Pan American Health Organization, which reported that working in agriculture increases the risk of CKDnt, though significance was reached only when cross-sectional studies were excluded [46]. Additionally, our study identifies elevated ambient temperature as a significant moderator for CKD. This temperature-related risk factor has not been extensively explored in previous meta-analyses, highlighting the novel contribution of our research. In most studies, heat exposure was typically assessed using proxies such as outdoor work or residence in high ambient temperature areas, without direct workplace measurements or personal heat strain indices. To address the challenge of missing temperature data, this study used latitude as a proxy for ambient temperature. This approach not only minimizes bias from incomplete data but has also been previously employed to establish a causal relationship between ambient temperature and vaccine efficacy [47]. Similarly, agrochemical exposure was primarily estimated through self-reported questionnaire data rather than objective environmental sampling or biomonitoring of pesticide metabolites in biological specimens (e.g., urine or blood). Recognizing these challenges, we emphasize the need for future research to incorporate real-time environmental monitoring and biomarker assessments to improve exposure characterization and strengthen causal inferences.

Heat stress associated with repetitive dehydration has been shown to be the major initiating and prognostic factor for CKDnt. Two widely discussed aspects of repetitive dehydration responsible for the initiation of CKDnt are antioxidant enzyme activity and osmolarity change [48,49]. Some animal studies have shown that chronic heat stress caused over-expression of antioxidant enzymes [48], and increased oxidative stress [50]. In addition, hyperosmolarity caused by heat stress activates vasopressin, which increases water absorption, leading to vasoconstriction, both of which cause ischemic kidney injury [49]. Furthermore, increased fructose, fructokinase, and reactive oxygen species (ROS) involved in the polyol pathway have been associated with CKDnt [51–53]. In this review, we noted that few studies provided ambient temperatures and amount of fluid intake during the study period for comparison and further analysis. We recommend that future relevant epidemiological studies need to provide the ambient temperature and fluid intake during the study period.

Drinking well water may increase the risk of CKD in Sri Lanka. Previous epidemiological studies have generally assumed that drinking well water is associated with a higher risk of heavy metal exposure. One included analysis showed that CKD occurred in areas where the groundwater was the main source of drinking water [5]. However, studies analysing several drinking water samples in India found the opposite result. The levels of the major ions and trace elements in these samples were within the recommended limits and were unlikely to be nephrotoxic [54]. As few studies have investigated heavy metal levels in well water, future epidemiological studies should measure heavy metals in blood or urine to better elucidate the association between heavy metal contamination and CKD.

Infectious diseases may be associated with CKD in agricultural populations, such as leptospirosis [55–58], hantavirus [56,58], chikungunya [59], and malaria [60] in tropical countries. In a mountainous area of Taiwan, a typhoon and rainstorm caused a re-emerging of leptospirosis, resulting in a regional CKDnt epidemic [55]. However, an investigation of CKDnt in a Nicaraguan mining community provided evidence against the hypotheses that leptospirosis or hantavirus can lead to CKDnt due to unclear timing of infection and CKDnt onset [56]. Future studies should design prospective cohort studies with baseline serologic surveys in susceptible communities, tracking changes in the prevalence of these infectious diseases and CKDnt after a climate disaster to clarify the impact of infectious diseases on CKDnt.

Drinking well water and exposure to agrochemicals are significant risk factors for CKD in Sri Lanka, underscoring the multifactorial nature of CKDnt in the region and suggesting that elevated temperature may not be the sole contributor. Although some of the included studies have shown that agrochemicals are a risk factor for CKD [26,28,61], none of the included studies provided environmental monitoring or urine and/or serum metabolite data, and agrochemical use relied solely on questionnaire data. In our review, we were only able to obtain qualitative material (yes/no) from one-third of the included studies. Pesticides and herbicides are widely used in agriculture [11,34], and some are nephrotoxic [27]. The chemical content of agrochemicals is complex, and misuse is common. Exposure to agrochemicals includes the direct route by dermal contact and inhalation and the indirect route by ingestion of contaminated food. Further research should focus on incorporating the habit of drinking sugary drinks into epidemiological studies to better understand its potential impact on CKD progression.

Among agricultural workers in epidemic regions, a lower BMI was identified as a risk factor for CKD—a finding that contrasts with previous studies linking obesity to the disease [40,62]. This phenomenon suggests that CKDnt is less common among obese individuals and more prevalent among agricultural workers engaged in physically demanding labor. Moreover, given that low water intake was identified as a significant risk factor (pooled OR = 2.06, 95% CI 1.17–3.63), we propose that rehydration education is essential for preventing CKDnt in these workers.

Alcohol consumption is a significant risk factor for CKD in regions affected by CKDnt. In Taiwan, we observed that farmers who consumed alcoholic beverages while working experienced elevated core temperatures, which in turn led to acute kidney injury. In Central America, the consumption of illicit alcohol known as “lija”—often prepared and stored in repurposed industrial metal containers formerly used for pesticides—raises concerns about exposure to nephrotoxins such as lead and other heavy metals [25]. We recommend further research to obtain detailed data on alcohol consumption among agricultural workers to better understand its effects.

Our study found a positive association between family history and CKD. Some studies have suggested that family members share similar environmental risks. However, some studies have suggested that some genes are associated with susceptibility to kidney injury [5,16]. We recommend occupational health management for employees with a family history of CKD.

Despite the common belief that farmers use NSAIDs more frequently than other populations and that this habit may increase the risk of CKD, this study shows that NSAIDs are not a significant risk factor for CKD in agricultural workers. It should be noted that the association between NSAIDs and CKD varied considerably between studies [30]. The definition of NSAID use also varied between the included studies, and the duration of exposure to these nephrotoxic drugs could not be accurately assessed in cross-sectional studies [29]. Therefore, the results should be interpreted with caution.

Some studies have suggested that the consumption of fructose-containing beverages accelerates the progression of CKD [63–65]. However, current epidemiological studies are still limited, and our review cannot conclude that the habit of drinking sugary drinks increases the risk of CKD. Further research should focus on incorporating the habit of drinking sugary drinks into epidemiological studies to better understand its potential impact on CKD progression.

### Limitation

One major limitation in current CKDnt research is the insufficient direct data on heat stress exposure. While ambient temperature has been used as a proxy for assessing heat-related risk, few studies have collected detailed workplace measurements, including Wet Bulb Globe Temperature (WBGT) or personal heat strain indices. The absence of these data restricts our ability to establish a direct causal link between heat stress and CKDnt progression. Future studies should prioritize real-time environmental monitoring to better quantify individual heat exposure and its physiological impacts. Similarly, research on agrochemical exposure is hindered by reliance on self-reported data rather than objective measurements. Most studies assess exposure based on questionnaire responses, lacking direct environmental sampling or biomonitoring of pesticide metabolites in biological specimens such as urine or blood. Given the potential nephrotoxicity of specific agrochemicals, standardized environmental and biological monitoring protocols are essential for a more accurate assessment of exposure risks. Addressing these study gaps is crucial for advancing our understanding of CKDnt etiology and developing targeted prevention strategies. Future research efforts should integrate environmental and occupational health assessments to refine exposure characterization and inform effective interventions. Our findings underscore the need for future research incorporating direct measurements of heat stress and occupational conditions. While our study establishes rising temperatures as a significant risk factor, precise physiological and environmental data—such as core body temperature, sweat rate, and workplace microclimate conditions—are essential for quantifying individual heat exposure and its cumulative effects on kidney function. Additionally, assessing real-time occupational conditions, including workload intensity, hydration practices, and protective measures, can provide crucial insights into how workplace factors contribute to CKDnt risk. In cross-sectional study protocols, studies can only use a single eGFR to determine the prevalence of CKD, rather than repeated measurements at intervals of at least three months, and this may not correspond to the chronicity standard for CKD. This practice may lead to an overestimation of the prevalence of CKD and a misclassified control group to the CKD case group, thereby influencing the OR toward the null value. To estimate the effect of risk factors, we did a meta-analysis for each risk factor independently and did not include all these factors at the same time because many factors are highly correlated. Repeated measurement with wearable heat monitors and biomarkers of kidney injury would help clarify causal pathways and refine targeted interventions. Future research should also explore interactions between chronic diseases, heat stress, and agrochemical exposure, particularly among agricultural workers and other vulnerable populations in tropical regions.

## Conclusions

Our findings establish CKDnt as a multifactorial tropical disease influenced by heat exposure, physically demanding work, and inadequate hydration. In addition, water contamination and agrochemical exposure appear to play significant roles in CKD development in Sri Lanka, emphasizing the need for clean drinking water and stricter agrochemical regulations. Addressing these issues is critical for creating effective occupational health policies and tailored prevention programs to reduce CKDnt among high-risk agricultural populations in tropical endemic regions.

## Supporting information

S1 Text Additional information on the methods and the results. Table A. PRISMA 2020 Checklist. Table B. Search strategy. Table C. Detailed characteristics of included studies. Table D. Meta-regression results for latitude on CKD. Table E. Meta-regression results for temperature on CKD. Fig A. Funnel plot of the odds ratio for CKD. Fig B. Risk of information bias.
(DOCX)
