# Supplementary material for: Environmental risk factors for chronic kidney disease of non-traditional causes in tropical coastal areas: A systematic review and meta-analysis
Source: PLoS Negl Trop Dis. 2025 May 6;19(5):e0013056. doi: 10.1371/journal.pntd.0013056 (PMC12054882; doi:10.1371/journal.pntd.0013056)
Supplement: S1 Text — Table A. PRISMA 2020 Checklist. Table B. Search strategy. Table C. Detailed characteristics of included studies. Table D. Meta-regression results for latitude on CKD. Table E. Meta-regression results for temperature on CKD. Fig A. Funnel plot of the odds ratio for CKD. Fig B. Risk of information bias. (DOCX) [file pntd.0013056.s001.docx]

**Environmental Risk Factors for Chronic Kidney Disease of Non-Traditional Causes in Tropical Coastal Areas: A Systematic Review and Meta-Analysis**

**Table A.** PRISMA 2020 Checklist

| **Section and Topic** | **Item #** | **Checklist item** | **Location where item is reported** |
| --- | --- | --- | --- |
| **TITLE** | | |  |
| Title | 1 | Identify the report as a systematic review. | P1 |
| **ABSTRACT** | | |  |
| Abstract | 2 | See the PRISMA 2020 for Abstracts checklist. | P2 |
| **INTRODUCTION** | | |  |
| Rationale | 3 | Describe the rationale for the review in the context of existing knowledge. | P4 |
| Objectives | 4 | Provide an explicit statement of the objective(s) or question(s) the review addresses. | P4 |
| **METHODS** | | |  |
| Eligibility criteria | 5 | Specify the inclusion and exclusion criteria for the review and how studies were grouped for the syntheses. | P6 |
| Information sources | 6 | Specify all databases, registers, websites, organisations, reference lists and other sources searched or consulted to identify studies. Specify the date when each source was last searched or consulted. | P8 |
| Search strategy | 7 | Present the full search strategies for all databases, registers and websites, including any filters and limits used. | P8 |
| Selection process | 8 | Specify the methods used to decide whether a study met the inclusion criteria of the review, including how many reviewers screened each record and each report retrieved, whether they worked independently, and if applicable, details of automation tools used in the process. | P8 |
| Data collection process | 9 | Specify the methods used to collect data from reports, including how many reviewers collected data from each report, whether they worked independently, any processes for obtaining or confirming data from study investigators, and if applicable, details of automation tools used in the process. | P9 |
| Data items | 10a | List and define all outcomes for which data were sought. Specify whether all results that were compatible with each outcome domain in each study were sought (e.g. for all measures, time points, analyses), and if not, the methods used to decide which results to collect. | P9 |
|  | 10b | List and define all other variables for which data were sought (e.g. participant and intervention characteristics, funding sources). Describe any assumptions made about any missing or unclear information. | P9 |
| Study risk of bias assessment | 11 | Specify the methods used to assess risk of bias in the included studies, including details of the tool(s) used, how many reviewers assessed each study and whether they worked independently, and if applicable, details of automation tools used in the process. | P10 |
| Effect measures | 12 | Specify for each outcome the effect measure(s) (e.g. risk ratio, mean difference) used in the synthesis or presentation of results. | P11 |
| Synthesis methods | 13a | Describe the processes used to decide which studies were eligible for each synthesis (e.g. tabulating the study intervention characteristics and comparing against the planned groups for each synthesis (item #5)). | P11 |
|  | 13b | Describe any methods required to prepare the data for presentation or synthesis, such as handling of missing summary statistics, or data conversions. | P11 |
|  | 13c | Describe any methods used to tabulate or visually display results of individual studies and syntheses. | P11 |
|  | 13d | Describe any methods used to synthesize results and provide a rationale for the choice(s). If meta-analysis was performed, describe the model(s), method(s) to identify the presence and extent of statistical heterogeneity, and software package(s) used. | P11 |
|  | 13e | Describe any methods used to explore possible causes of heterogeneity among study results (e.g. subgroup analysis, meta-regression). | P11 |
|  | 13f | Describe any sensitivity analyses conducted to assess robustness of the synthesized results. | P11 |
| Reporting bias assessment | 14 | Describe any methods used to assess risk of bias due to missing results in a synthesis (arising from reporting biases). | P11 |
| Certainty assessment | 15 | Describe any methods used to assess certainty (or confidence) in the body of evidence for an outcome. | P12 |
| **RESULTS** | | |  |
| Study selection | 16a | Describe the results of the search and selection process, from the number of records identified in the search to the number of studies included in the review, ideally using a flow diagram. | P12 |
|  | 16b | Cite studies that might appear to meet the inclusion criteria, but which were excluded, and explain why they were excluded. | P12 |
| Study characteristics | 17 | Cite each included study and present its characteristics. | P12 |
| Risk of bias in studies | 18 | Present assessments of risk of bias for each included study. | Supplementary Figure 2 |
| Results of individual studies | 19 | For all outcomes, present, for each study: (a) summary statistics for each group (where appropriate) and (b) an effect estimate and its precision (e.g. confidence/credible interval), ideally using structured tables or plots. | Table 1 |
| Results of syntheses | 20a | For each synthesis, briefly summarise the characteristics and risk of bias among contributing studies. | Supplementary Table 4 |
|  | 20b | Present results of all statistical syntheses conducted. If meta-analysis was done, present for each the summary estimate and its precision (e.g. confidence/credible interval) and measures of statistical heterogeneity. If comparing groups, describe the direction of the effect. | P12 |
|  | 20c | Present results of all investigations of possible causes of heterogeneity among study results. | P13 |
|  | 20d | Present results of all sensitivity analyses conducted to assess the robustness of the synthesized results. | P13 |
| Reporting biases | 21 | Present assessments of risk of bias due to missing results (arising from reporting biases) for each synthesis assessed. | P14 |
| Certainty of evidence | 22 | Present assessments of certainty (or confidence) in the body of evidence for each outcome assessed. | P14 |
| **DISCUSSION** | | |  |
| Discussion | 23a | Provide a general interpretation of the results in the context of other evidence. | P15 |
|  | 23b | Discuss any limitations of the evidence included in the review. | P19 |
|  | 23c | Discuss any limitations of the review processes used. | P19 |
|  | 23d | Discuss implications of the results for practice, policy, and future research. | P19 |
| **OTHER INFORMATION** | | |  |
| Registration and protocol | 24a | Provide registration information for the review, including register name and registration number, or state that the review was not registered. | NA |
|  | 24b | Indicate where the review protocol can be accessed, or state that a protocol was not prepared. | NA |
|  | 24c | Describe and explain any amendments to information provided at registration or in the protocol. | NA |
| Support | 25 | Describe sources of financial or non-financial support for the review, and the role of the funders or sponsors in the review. | P34 |
| Competing interests | 26 | Declare any competing interests of review authors. | P34 |
| Availability of data, code and other materials | 27 | Report which of the following are publicly available and where they can be found: template data collection forms; data extracted from included studies; data used for all analyses; analytic code; any other materials used in the review. | P22 |

**Table B.** Search strategy

| Online database | Number | Search strategy |
| --- | --- | --- |
| Pubmed | 510 | “((((CKDu[tw] OR CKDnt[tw] OR CINAC[tw] OR mesoamerican nephropath*[tw] OR meso american nephropath*[tw] OR "Uddanam Nephropathy"[tw] OR "Sri Lankan Nephropathy"[tw] OR ((chronic kidney disease*[tw] OR "Renal Insufficiency, Chronic"[Mesh]) AND (uncertain etiolog*[tw] OR unknown etiolog*[tw] OR undetermined etiolog*[tw] OR uncertain aetiolog*[tw] OR unknown aetiolog*[tw] OR undetermined aetiolog*[tw] OR non-traditional cause*[tw] OR nontraditional cause*[tw] OR uncertain cause*[tw] OR unknown cause*[tw] OR undetermined cause*[tw])) OR ((CKD[ti] OR chronic kidney disease*[ti]) AND (unknown[ti] OR uncertain[ti] OR undetermined[ti] OR non-traditional*[ti] OR nontraditional*[ti])))) AND (Sri Lanka*[tw] OR "India"[tw] OR "Sri Lanka"[Mesh] OR "India"[Mesh] OR "Colombo"[tw] OR "Delhi"[tw] OR "Mumbai"[tw] OR "Bangalore"[tw] OR "Hyderabad"[tw] OR Mexico[tw] OR Mexican*[tw] OR Nicaragua*[tw] OR Guatemala*[tw] OR El Salvador*[tw] OR Costa Rica*[tw] OR Panama*[tw] OR Belize[tw] OR Hondura*[tw] OR Latin America*[tw] OR Central America*[tw] OR "Latin America"[Mesh] OR "Central America"[Mesh] OR "Mexico"[Mesh] OR "Nicaragua"[Mesh] OR "Guatemala"[Mesh] OR "El Salvador"[Mesh] OR "Costa Rica"[Mesh] OR "Panama"[Mesh] OR "Belize"[Mesh] OR "Honduras"[Mesh] OR mesoamerica*[tw] OR meso america*[tw] OR "South America"[Mesh] OR South America*[tw] OR Argentina*[tw] OR Bolivia*[tw] OR Brazil*[tw] OR Chile*[tw] OR Colombia*[tw] OR Ecuador*[tw] OR French Guiana*[tw] OR Guyana*[tw] OR Paraguay*[tw] OR Peru*[tw] OR Suriname[tw] OR Uruguay*[tw] OR Venezuela*[tw] OR "Asia"[Mesh] OR "Asia"[tw] OR (Asian*[tw] NOT Asian American*[tw]) OR Kazakhstan[tw] OR Kyrgyzstan[tw] OR Tajikistan[tw] OR Turkmenistan[tw] OR Uzbekistan[tw] OR Russia*[tw] OR Borneo[tw] OR Brunei[tw] OR Cambodia*[tw] OR Indonesia*[tw] OR Laos[tw] OR Malaysia*[tw] OR Myanmar[tw] OR Philippines[tw] OR  Singapore[tw] OR Thailand[tw] OR Timor-Leste[tw] OR Vietnam*[tw] OR Bangladesh*[tw] OR Bhutan[tw] OR Nepal*[tw] OR Pakistan*[tw] OR China[tw] OR Chinese[tw] OR Hong Kong[tw] OR Tibet[tw] OR Japan*[tw] OR Korea*[tw] OR Mongolia*[tw] OR Taiwan*[tw] OR Middle East*[tw] OR Afghanistan*[tw] OR Bahrain[tw] OR Iran*[tw] OR Iraq*[tw] OR Israel*[tw] OR Jordan*[tw] OR Kuwait*[tw] OR Lebanon[tw] OR Lebanese[tw] OR Oman[tw] OR Qatar[tw] OR Saudi Arabia*[tw] OR Syria*[tw] OR Turkey[tw] OR United Arab Emirates[tw] OR Yemen*[tw])) AND ("2000/01/01"[Date - Publication] : "2024/12/31"[Date - Publication]) AND (English[lang]) NOT ("Comment"[Publication Type] OR "Letter"[Publication Type] OR "Editorial"[Publication Type] OR ("Animals"[Mesh] NOT "Humans"[Mesh]))” |
| Embase | 403 | ('ckdu':ti,ab OR 'ckdnt':ti,ab OR 'cinac':ti,ab OR 'chronic interstitial nephritis in agricultural communities':ti,ab OR  'mesoamerican nephropathy'/exp OR 'mesoamerican nephropath*':ti,ab OR 'meso american nephropath*':ti,ab OR ('Uddanam Nephropathy':ti,ab OR 'Sri Lankan Nephropathy':ti,ab) OR (('chronic kidney disease*':ti,ab OR 'chronic kidney failure'/exp) AND ('uncertain etiolog*':ti,ab OR 'unknown etiolog*':ti,ab OR 'undetermined etiolog*':ti,ab OR 'uncertain aetiolog*':ti,ab OR 'unknown aetiolog*':ti,ab OR 'undetermined aetiolog*':ti,ab OR 'non-traditional cause*':ti,ab OR 'nontraditional cause*':ti,ab OR 'uncertain cause*':ti,ab OR 'unknown cause*':ti,ab OR 'undetermined cause*':ti,ab OR 'idiopathic disease'/exp)) OR (('ckd':ti OR 'chronic kidney disease*':ti) AND  ('unknown':ti OR 'uncertain':ti OR 'undetermined':ti OR 'non-traditional*':ti OR 'nontraditional*':ti))) AND  ('sri lanka*':ti,ab OR 'india':ti,ab OR 'sri lanka'/exp OR 'sri lankan'/exp OR 'india'/exp OR 'indian'/exp OR  'colombo':ti,ab OR 'delhi':ti,ab OR 'mumbai':ti,ab OR 'bangalore':ti,ab OR 'hyderabad':ti,ab OR 'mexico':ti,ab OR 'mexican*':ti,ab OR 'nicaragua*':ti,ab OR 'guatemala*':ti,ab OR 'el salvador*':ti,ab OR 'costa rica*':ti,ab OR 'panama*':ti,ab OR 'belize':ti,ab OR 'hondura*':ti,ab OR 'latin america*':ti,ab OR 'central america*':ti,ab OR 'central america'/exp OR 'mexico'/exp OR 'nicaragua'/exp OR 'guatemala'/exp OR 'el salvador'/exp OR 'costa rica'/exp OR 'panama'/exp OR 'belize'/exp OR 'honduras'/exp OR 'mesoamerica*':ti,ab OR 'meso america*':ti,ab OR 'south america'/exp OR 'south america':ti,ab OR 'argentina*':ti,ab OR 'bolivia*':ti,ab OR 'brazil*':ti,ab OR 'chile*':ti,ab OR 'colombia*':ti,ab OR 'ecuador*':ti,ab OR 'french guiana*':ti,ab OR 'guyana*':ti,ab OR 'paraguay*':ti,ab OR 'peru*':ti,ab OR 'suriname':ti,ab OR 'uruguay*':ti,ab OR 'venezuela*':ti,ab OR 'asia'/exp OR 'asia':ti,ab OR  (asian*:ti,ab NOT 'asian american*':ti,ab) OR kazakhstan':ti,ab OR 'kyrgyzstan':ti,ab OR tajikistan':ti,ab OR 'turkmenistan':ti,ab OR 'uzbekistan':ti,ab OR 'russia*':ti,ab OR 'borneo':ti,ab OR 'brunei':ti,ab OR 'cambodia*':ti,ab OR 'indonesia*':ti,ab OR 'laos':ti,ab OR 'malaysia*':ti,ab OR 'myanmar':ti,ab OR 'philippines':ti,ab OR 'singapore':ti,ab OR 'thailand':ti,ab OR 'timor-leste':ti,ab OR 'vietnam*':ti,ab OR 'bangladesh*':ti,ab OR 'bhutan':ti,ab OR 'nepal*':ti,ab OR 'pakistan*':ti,ab OR 'china':ti,ab OR 'chinese':ti,ab OR 'hong kong':ti,ab OR 'tibet':ti,ab OR 'japan*':ti,ab OR  'korea*':ti,ab OR 'mongolia*':ti,ab OR 'taiwan*':ti,ab OR 'middle east*':ti,ab OR 'afghanistan*':ti,ab OR 'bahrain':ti,ab OR 'iran*':ti,ab OR 'iraq*':ti,ab OR 'israel*':ti,ab OR 'jordan*':ti,ab OR 'kuwait*':ti,ab OR  'lebanon':ti,ab OR 'lebanese':ti,ab OR 'oman':ti,ab OR 'qatar':ti,ab OR 'saudi arabia*':ti,ab OR 'syria*':ti,ab OR 'turkey*':ti,ab OR 'united arab emirates':ti,ab OR 'yemen*':ti,ab) AND [2000-2024]/py AND english]/lim  AND [embase]/lim NOT (comment*:ti OR letter:it OR editorial:it OR 'editorial'/exp OR 'letter'/exp OR  [conference abstract]/lim OR [editorial]/lim OR [letter]/lim OR ('animal'/exp NOT 'human'/exp)) |
| Web of Science | 1156 | TS=("CKDu" OR "CKDnt" OR "CINAC" OR "Mesoamerican Nephropathy" OR "Meso American Nephropathy" OR "Uddanam Nephropathy" OR "Sri Lankan Nephropathy" OR (("Chronic Kidney Disease*" OR "Chronic Renal Insufficiency") AND ("Uncertain Etiology" OR "Unknown Etiology" OR "Undetermined Etiology" OR "Uncertain Aetiology" OR "Unknown Aetiology" OR "Undetermined Aetiology" OR "Non-Traditional Cause*" OR "Nontraditional Cause*" OR "Uncertain Cause*" OR "Unknown Cause*" OR "Undetermined Cause*")) OR (("CKD" OR "Chronic Kidney Disease*") AND ("Unknown" OR "Uncertain" OR "Undetermined" OR "Non-Traditional*" OR "Nontraditional*"))) AND TS=("Sri Lanka*" OR "India" OR "Colombo" OR "Delhi" OR "Mumbai" OR "Bangalore" OR "Hyderabad" OR "Mexico" OR "Mexican*" OR "Nicaragua" OR "Guatemala" OR "El Salvador" OR "Costa Rica" OR "Panama" OR "Belize" OR "Honduras" OR "Latin America" OR "Central America" OR "Mesoamerica*" OR "Meso America*" OR "South America" OR "Argentina" OR "Bolivia" OR "Brazil" OR "Chile" OR "Colombia" OR "Ecuador" OR "French Guiana" OR "Guyana" OR "Paraguay" OR "Peru" OR "Suriname" OR "Uruguay" OR "Venezuela" OR "Asia" OR ("Asian*" NOT "Asian American*") OR "Kazakhstan" OR "Kyrgyzstan" OR "Tajikistan" OR "Turkmenistan" OR "Uzbekistan" OR "Russia*" OR "Borneo" OR "Brunei" OR "Cambodia" OR "Indonesia" OR "Laos" OR "Malaysia" OR "Myanmar" OR "Philippines" OR "Singapore" OR "Thailand" OR "Timor-Leste" OR "Vietnam" OR "Bangladesh" OR "Bhutan" OR "Nepal" OR "Pakistan" OR "China" OR "Chinese" OR "Hong Kong" OR "Tibet" OR "Japan" OR "Korea" OR "Mongolia" OR "Taiwan" OR "Middle East" OR "Afghanistan" OR "Bahrain" OR "Iran" OR "Iraq" OR "Israel" OR "Jordan" OR "Kuwait" OR "Lebanon" OR "Oman" OR "Qatar" OR "Saudi Arabia" OR "Syria" OR "Turkey" OR "United Arab Emirates" OR "Yemen") AND PY=(2000-2024) AND LA=(English) NOT DT=("Comment" OR "Letter" OR "Editorial") |

**Table C.** Detailed characteristics of included studies

| **Authors, publication year, reference** | **Country** | **Sampling method** | | **Age** | **Male** | **Female** | **Outcome measurement** | **Predictor** | **Adjusted covariates** |
| --- | --- | --- | --- | --- | --- | --- | --- | --- | --- |
| 1. Sanoff et al. 2010 [1] | Nicaragua | health centres advertised screening | | >18 | 848 | 149 | single blood test | eGFR < 60 | age, gender, hypertension, diabetes, family history of CKD, BMI |
| 2. Torres, et al. 2010 [2] | Nicaragua | 5 villages, selected by convenience | | 20‒60 | 479 | 617 | single blood test | eGFR < 60 | age, hypertension, diabetes, BMI, NSAIDs, history of urinary tract infection, renal lithiasis |
| 3. Athuraliya^,^ et al. 2011 [3] | Sri Lanka | house-to-house screening in three distinct regions | | >19 | 2889 | 3264 | single blood test | proteinuric-CKD | age, gender, occupation, agrochemicals, family history, diabetes, hypertension |
| 4. O'Donnell, et al. 2011 [4] | Nicaragua | a random sample of households in 22 communities | | ≥18 | 298 | 473 | single blood test | eGFR < 60 | age and sex |
| 5. Orantes, et al. 2011 [5] | El Salvador | not mentioned | | ≥18 | 343 | 432 | single blood test | eGFR < 60 | age, sex, family history of CKD, agrochemical, hypertension, diabetes, BMI, dyslipidemia, metabolic syndrome |
| 6. Peraza, et al. 2012 [6] | El Salvador | not mentioned | | 20‒60 | 256 | 408 | single blood test | eGFR < 60 | age, smoking |
| 7. Nanayakkara, et al. 2014 [7] | Sri Lanka | randomly selected healthy males | | 16‒70 | 597 | 0 | biopsy-proven | cases who developed CKDu in the clinical course of tubulointerstitial damages | not mentioned |
| 8. Orantes, et al. 2014 [8] | \| El Salvador El Salvador \| Cross-sectional \| CKD \| \| --- \| --- \| --- \| | a door-to-door survey in agricultural communities | | ≥18 | 976 | 1412 | repeated blood tests for ≥ 3 months | eGFR < 60 | age, family history of CKD, diabetes, hypertension, tobacco use, alcohol use, medicinal plant use, NSAIDs use, agrochemicals, BMI, central obesity |
| 9. Raines, et al. 2014 [9] | Nicaragua | door-to-door canvassing in a community | | 15‒69 | 166 | 258 | single blood test | eGFR < 60 | age, sex, BMI, blood pressure, hypertension, diabetes, smoking, alcohol use, daily fructose, intake, NSAIDs use |
| 10. Vela, et al. 2014 [10] | El Salvador | not mentioned | | ≥15 | 110 | 113 | single blood test | eGFR < 60 | not mentioned |
| 11. Lebov, et al. 2015 [11] | Nicaragua | cluster random sampling | | 18‒70 | 949 | 1320 | single blood test | eGFR < 60 | age, sex, diabetes, high blood pressure |
|  |  |  | |  |  |  |  |  |  |
| 12. Jayasumana et al. 2015 [12] | Sri Lanka | hospital patients and community controls | | NA | 187 | 118 | Single blood test | Medical diagnosis at the hospital | sex, education, agrochemicals, drinking well water, smoking, alcohol, betel chewing, NSAIDs, family history of CKD |
| 13. Siriwardhana, et al. 2015 [13] | Sri Lanka | Random sampling from CKDnt patients of a hospital and community controls | | NA | 118 | 82 | Single blood test | Medical diagnosis at the hospital | agrochemicals, drinking well water, malaria, black tea, fresh water fish, low water intake |
| 14. Anand, et al 2019 [14] | Sri Lanka | persons referred to hospital nephrologists as new patients over 1 year | | ≥23 | 447 | 153 | biopsy-proven | tubulointerstitial kidney disease | age, education, birthplace, current residence, family history of any kidney disease, occupation, water source, ever use of well water, tobacco use, alcohol use, kidney stone, snake bite |
| 15. Orantes-Navarro, et al. 2019 [15] | El Salvador | two-stage cluster sample | | ≥20 | 1706 | 3111 | single blood test | eGFR < 60 and/or albumin-to-creatinine ratio > 30 mg/g | not mentioned |
| 16. Herrera-Valdés, et al. 2019 [16] | El Salvador | Not mentioned | | ≥18 | NA | NA | Not mentioned | Not mentioned | agrochemicals, consumption of river or well water |
| 17. Ruwanpathirana, et al. 2019 [17] | Sri Lanka | randomly sampling | | ≥18 | 219 | 969 | single blood test | eGFR < 60 | agrochemicals, smoking, alcohol, well water, amount of water consumed, work outside exposed to the sun |
| 18. Tatapudi, et al. 2019 [18] | India | randomly sampling | | ≥18 | 136 | 158 | single blood test | eGFR < 60 | age, gender, education, smoking, alcohol |
| 19. Ferguson, et al. 2020 [19] | Nicaragua | all eligible members of a sequential household | | 17‒102 | 501 | 642 | one point-of-care capillary creatinine–measuring system, | eGFR < 60 | sex, age, high blood pressure, diabetes |
| 20. Gummidi, et al. 2020 [20] | India | multistage cluster random sampling from 67 villages | | >18 | 1180 | 1222 | single blood test | eGFR < 60 | age, sex, education, income, outdoor worker, tobacco use, alcohol use, pain killer use, hypertension, diabetes, BMI, family history of CKD |
| 21. Aguilar-Ramirez, et al. 2021 [21] | Mexico | all adults aged 20-60 years in 3 rural communities were invited | | 20‒60 | 190 | 389 | single blood test | eGFR < 60 or albumin-to-creatinine ratio > 30 mg/g | age, sex, agriculture, family history of kidney disease, diabetes, hypertension, BMI |
| 22. Chang, et al. 2021 [22] | Taiwan | community-based integrated screening | | 15‒60 | 13943 | 24073 | single blood test | eGFR < 60 | age, sex, education, urbanization, BMI, comorbidities, use of Chinese herbs |
| 23. Miller, et al. 2021 [23] | Guatemala | randomly selected households | ≥18 | | 280 | 527 | repeated confirmatory blood test | eGFR < 60 | residence, age, poverty, the interaction between poverty and sugarcane employment, diabetes, BMI, tobacco use |
| 24. Chang, et al. 2023[24] | Taiwan | national cohort | >40 | | 2126418 | 2432267 | single blood test | eGFR < 60 | smoking, alcohol drinking, betel nut chewing, exercise, BMI, hyperlipidemia, heart disease, liver disease, gout, urolithiasis |
| 25. Figueroa-Solis, et al. 2023 [25] | Guatemala, Nicaragua | national representative sampling | ≥18 | | 4562 | 4470 | single blood test | eGFR < 60 | age, sex, education, income, length of employment, zone, physical demand, agrochemicals, NSIADs |
| 26. Sinha,et al. 2023 [46] | India | Hospital patients | All | | 138 | 60 | single blood test | eGFR < 60 | sex, smoking, salt intake, agrochemicals, drinking water, availability of shade |
| 27. Strasma, et al. 2023 [26] | Nicaragua | randomly selected households | 15‒59 | | 714 | 1081 | single blood test | eGFR < 60 | sex, age, smoking, hypertension, diabetes, NSAIDs, Malaria |
| 28. Gonzalez-Quiroz, et al. 2024 [27] | Nicaragua | Community-based cohort | 18‒30 | | 478 | 293 | single blood test | eGFR < 60 | hard physical effort, agrochemicals, smoking, alcohol, NSAIDs |

**Table D.** Meta-regression results for latitude on CKD

|  | Point  estimate | Standard  error | 95%  Lower | 95%  Upper | Z-value | p-Value |
| --- | --- | --- | --- | --- | --- | --- |
| Intercept | 1.0603 | 0.2462 | 0.5777 | 1.5428 | 4.3063 | <.0001 |
| Latitude | -0.0408 | 0.0128 | -0.0659 | -0.0156 | -3.1793 | 0.0015 |

Mixed-Effects Model (k = 31; tau^2^ estimator: REML)

tau^2^ (estimated amount of residual heterogeneity): 0.0103 (SE = 0.0245)

tau (square root of estimated tau^2^ value): 0.1016

*I^2^* (residual heterogeneity/unaccounted variability): 7.02%

*H^2^* (unaccounted variability/sampling variability): 1.08

*R^2^* (amount of heterogeneity accounted for): 80.39%

Test for Residual Heterogeneity: QE (df = 29) = 16.4806, p-val = 0.9696

Test of Moderators (coefficient 2): QM (df = 1) = 10.1082, p-val = 0.0015

**Table E.** Meta-regression results for temperature on CKD

|  | Point estimate | Standard  error | 95%  Lower | 95%  Upper | Z-value | p-Value |
| --- | --- | --- | --- | --- | --- | --- |
| Intercept | -1.50 | 0.91 | -3.28 | 0.27 | -1.66 | 0.10 |
| Temperature | 0.07 | 0.04 | 0.005 | 0.14 | 2.09 | 0.04 |

Mixed-Effects Model (k = 31; tau^2^ estimator: REML)

tau^2^ (estimated amount of residual heterogeneity): 0.0308 (SE = 0.04)

tau (square root of estimated tau^2^ value): 0.18

*I^2^* (residual heterogeneity / unaccounted variability): 15.83%

*H^2^* (unaccounted variability/sampling variability): 1.19

*R^2^* (amount of heterogeneity accounted for): 41.55%

Test for Residual Heterogeneity: QE (df = 29) = 20.2369, p-val = 0.899

Test of Moderators (coefficient 2): QM (df = 1) = 4.3609, p-val = 0.04

**
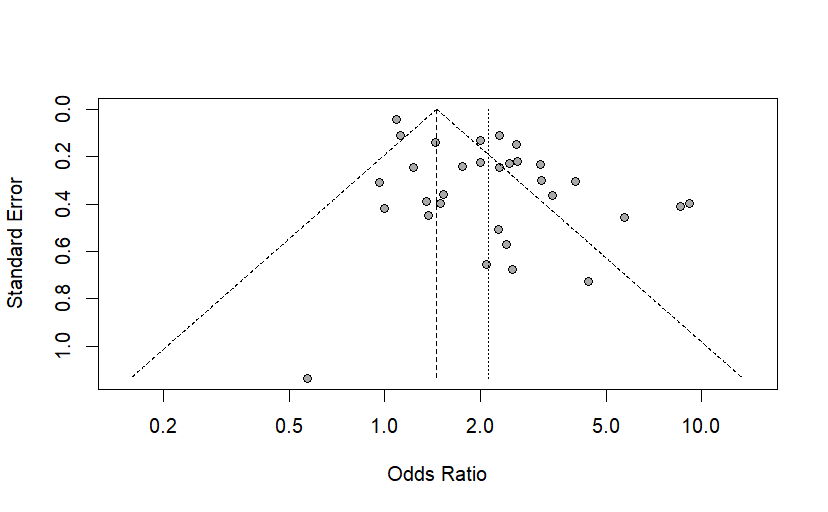
**

**Fig A.** Funnel plot of the odds ratio for CKD.


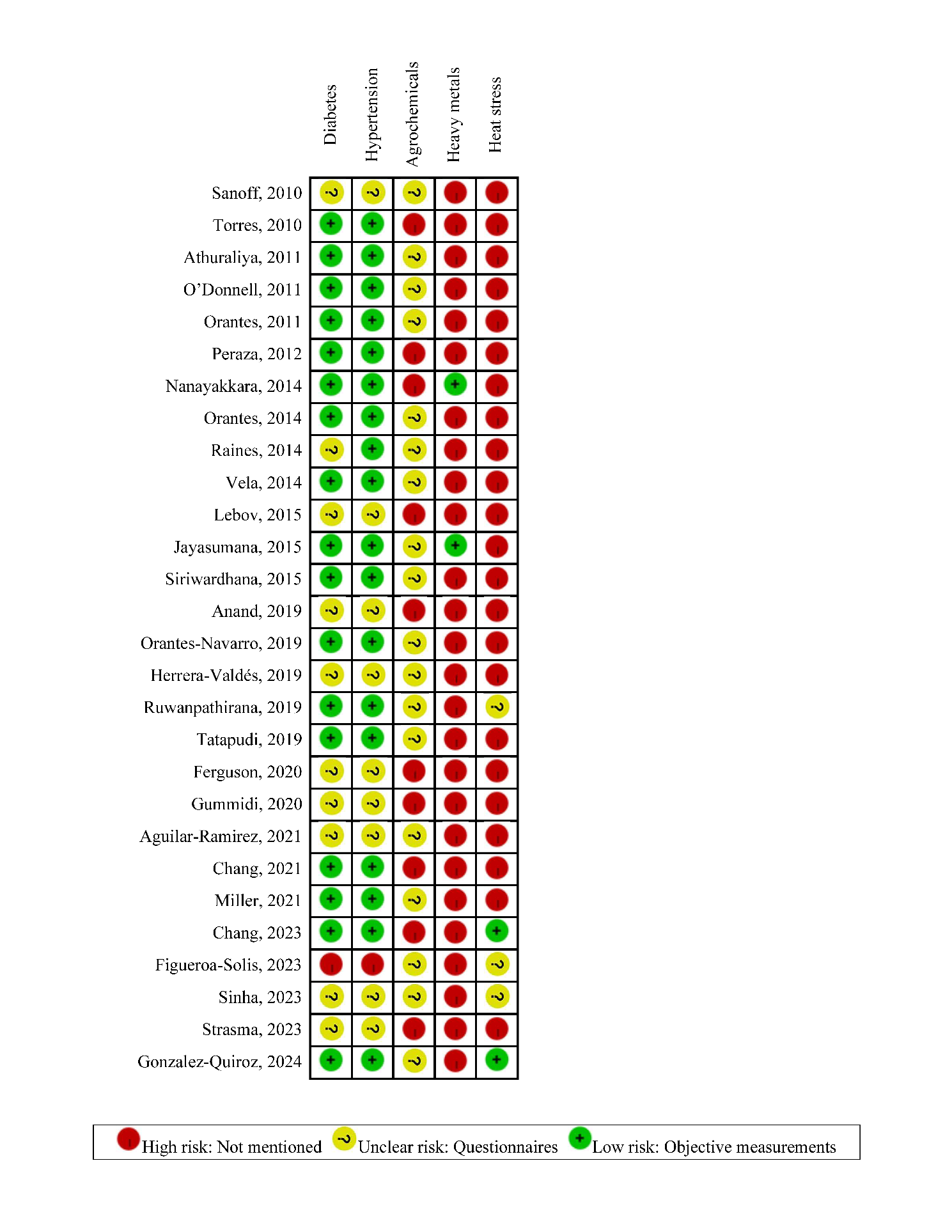
**Fig B.** Risk of information bias.

**References**

1. Sanoff SL, Callejas L, Alonso CD, Hu Y, Colindres RE, Chin H, et al. Positive association of renal insufficiency with agriculture employment and unregulated alcohol consumption in Nicaragua. Ren Fail. 2010;32(7):766-77. Epub 2010/07/29. doi: 10.3109/0886022X.2010.494333. PubMed PMID: 20662688; PubMed Central PMCID: PMCPMC3699859.

2. Torres C, Aragón A, González M, López I, Jakobsson K, Elinder CG, et al. Decreased kidney function of unknown cause in Nicaragua: a community-based survey. Am J Kidney Dis. 2010;55(3):485-96. Epub 20100208. doi: 10.1053/j.ajkd.2009.12.012. PubMed PMID: 20116154.

3. Athuraliya NT, Abeysekera TD, Amerasinghe PH, Kumarasiri R, Bandara P, Karunaratne U, et al. Uncertain etiologies of proteinuric-chronic kidney disease in rural Sri Lanka. Kidney Int. 2011;80(11):1212-21. Epub 2011/08/13. doi: 10.1038/ki.2011.258. PubMed PMID: 21832982.

4. O'Donnell JK, Tobey M, Weiner DE, Stevens LA, Johnson S, Stringham P, et al. Prevalence of and risk factors for chronic kidney disease in rural Nicaragua. Nephrol Dial Transplant. 2011;26(9):2798-805. Epub 2010/07/10. doi: 10.1093/ndt/gfq385. PubMed PMID: 20615905; PubMed Central PMCID: PMCPMC4592358.

5. Orantes CM, Herrera R, Almaguer M, Brizuela EG, Hernández CE, Bayarre H, et al. Chronic kidney disease and associated risk factors in the Bajo Lempa region of El Salvador: Nefrolempa study, 2009. MEDICC Rev. 2011;13(4):14-22. Epub 2011/12/07. doi: 10.37757/mr2011v13.N4.5. PubMed PMID: 22143603.

6. Peraza S, Wesseling C, Aragon A, Leiva R, Garcia-Trabanino RA, Torres C, et al. Decreased Kidney Function Among Agricultural Workers in El Salvador. American Journal of Kidney Diseases. 2012;59(4):531-40. doi: 10.1053/j.ajkd.2011.11.039. PubMed PMID: WOS:000302117500323.

7. Nanayakkara S, Senevirathna ST, Abeysekera T, Chandrajith R, Ratnatunga N, Gunarathne ED, et al. An integrative study of the genetic, social and environmental determinants of chronic kidney disease characterized by tubulointerstitial damages in the North Central Region of Sri Lanka. J Occup Health. 2014;56(1):28-38. Epub 20131218. doi: 10.1539/joh.13-0172-oa. PubMed PMID: 24351856.

8. Orantes CM, Herrera R, Almaguer M, Brizuela EG, Nunez L, Alvarado NP, et al. Epidemiology of chronic kidney disease in adults of Salvadoran agricultural communities. MEDICC Rev. 2014;16(2):23-30. Epub 2014/06/01. doi: 10.37757/MR2014.V16.N2.5. PubMed PMID: 24878646.

9. Raines N, Gonzalez M, Wyatt C, Kurzrok M, Pool C, Lemma T, et al. Risk Factors for Reduced Glomerular Filtration Rate in a Nicaraguan Community Affected by Mesoamerican Nephropathy. Medicc Review. 2014;16(2):16-22. PubMed PMID: WOS:000347507900004.

10. Vela XF, Henríquez DO, Zelaya SM, Granados DV, Hernández MX, Orantes CM. Chronic kidney disease and associated risk factors in two Salvadoran farming communities, 2012. MEDICC Rev. 2014;16(2):55-60. Epub 2014/06/01. doi: 10.37757/mr2014.V16.N2.9. PubMed PMID: 24878650.

11. Lebov JF, Valladares E, Peña R, Peña EM, Sanoff SL, Cisneros EC, et al. A population-based study of prevalence and risk factors of chronic kidney disease in León, Nicaragua. Can J Kidney Health Dis. 2015;2:6. Epub 20150224. doi: 10.1186/s40697-015-0041-1. PubMed PMID: 25926994; PubMed Central PMCID: PMCPMC4414463.

12. Jayasumana C, Paranagama P, Agampodi S, Wijewardane C, Gunatilake S, Siribaddana S. Drinking well water and occupational exposure to Herbicides is associated with chronic kidney disease, in Padavi-Sripura, Sri Lanka -No section. Environmental Health: A Global Access Science Source. 2015;14(1). doi: 10.1186/1476-069X-14-6.

13. Siriwardhana EARIE, Perera PAJ, Sivakanesan R, Abeysekara T, Nugegoda D, Jayaweera JAAS. Dehydration and malaria augment the risk of developing chronic kidney disease in Sri Lanka. Indian Journal of Nephrology. 2015;25(3):146-51. doi: 10.4103/0971-4065.140712.

14. Anand S, Montez-Rath ME, Adasooriya D, Ratnatunga N, Kambham N, Wazil A, et al. Prospective Biopsy-Based Study of CKD of Unknown Etiology in Sri Lanka. Clin J Am Soc Nephrol. 2019;14(2):224-32. Epub 20190118. doi: 10.2215/cjn.07430618. PubMed PMID: 30659059; PubMed Central PMCID: PMCPMC6390926.

15. Orantes-Navarro CM, Almaguer-López MM, Alonso-Galbán P, Díaz-Amaya M, Hernández S, Herrera-Valdés R, et al. The Chronic Kidney Disease Epidemic in El Salvador: A Cross-Sectional Study. MEDICC Rev. 2019;21(2-3):29-37. doi: 10.37757/mr2019.V21.N2-3.7. PubMed PMID: 31373582.

16. Herrera-Valdés R, Almaguer-López MA, Orantes-Navarro CM, López-Marín L, Brizuela-Díaz EG, Bayarre-Vea H, et al. Epidemic of chronic kidney disease of nontraditional etiology in El Salvador: Integrated health sector action and south-south cooperation. MEDICC Review. 2019;21(3):46-52.

17. Ruwanpathirana T, Senanayake S, Gunawardana N, Munasinghe A, Ginige S, Gamage D, et al. Prevalence and risk factors for impaired kidney function in the district of Anuradhapura, Sri Lanka: a cross-sectional population-representative survey in those at risk of chronic kidney disease of unknown aetiology. BMC Public Health. 2019;19(1):763. Epub 20190614. doi: 10.1186/s12889-019-7117-2. PubMed PMID: 31200694; PubMed Central PMCID: PMCPMC6570843.

18. Tatapudi RR, Rentala S, Gullipalli P, Komarraju AL, Singh AK, Tatapudi VS, et al. High Prevalence of CKD of Unknown Etiology in Uddanam, India. Kidney Int Rep. 2019;4(3):380-9. Epub 20181016. doi: 10.1016/j.ekir.2018.10.006. PubMed PMID: 30899865; PubMed Central PMCID: PMCPMC6409405.

19. Ferguson R, Leatherman S, Fiore M, Minnings K, Mosco M, Kaufman J, et al. Prevalence and Risk Factors for CKD in the General Population of Southwestern Nicaragua. J Am Soc Nephrol. 2020;31(7):1585-93. Epub 2020/05/31. doi: 10.1681/asn.2019050521. PubMed PMID: 32471819; PubMed Central PMCID: PMCPMC7350996.

20. Gummidi B, John O, Ghosh A, Modi GK, Sehgal M, Kalra OP, et al. A Systematic Study of the Prevalence and Risk Factors of CKD in Uddanam, India. Kidney Int Rep. 2020;5(12):2246-55. Epub 20201016. doi: 10.1016/j.ekir.2020.10.004. PubMed PMID: 33305118; PubMed Central PMCID: PMCPMC7710882.

21. Aguilar-Ramirez D, Rana-Custodio A, Villa A, Rubilar X, Olvera N, Escobar A, et al. Decreased kidney function and agricultural work: a cross-sectional study in middle-aged adults from Tierra Blanca, Mexico. Nephrology Dialysis Transplantation. 2021;36(6):1030-8. doi: 10.1093/ndt/gfaa041. PubMed PMID: WOS:000704006300015.

22. Chang JC-J, Yang H-Y. Epidemiology of chronic kidney disease of undetermined aetiology in Taiwanese farmers: a cross-sectional study from Changhua Community-based Integrated Screening programme. Occupational and Environmental Medicine. 2021;78(12):849-58. doi: 10.1136/oemed-2021-107369.

23. Miller AC, Tuiz E, Shaw L, Flood D, Garcia P, Dhaenens E, et al. Population Estimates of GFR and Risk Factors for CKD in Guatemala. Kidney Int Rep. 2021;6(3):796-805. Epub 2021/03/19. doi: 10.1016/j.ekir.2020.12.015. PubMed PMID: 33732994; PubMed Central PMCID: PMCPMC7938058.

24. Chang CJY, H. Y. Chronic kidney disease among agricultural workers in Taiwann: a nationwide population-based study. Kidney International Reports. 2023. Epub Seotember 9, 2023. doi: <https://doi.org/10.1016/j.ekir.2023.09.004>.

25. Figueroa-Solis E, de Porras DGR, Rojas-Garbanzo M, Whitehead L, Zhang K, Delclos GL. Prevalence and Geographic Distribution of Self-Reported Chronic Kidney Disease and Potential Risk Factors in Central America. International Journal of Environmental Research and Public Health. 2023;20(2):14. doi: 10.3390/ijerph20021308. PubMed PMID: WOS:000914980100001.

26. Strasma A, Reyes ÁM, Aragón A, López I, Park LP, Hogan SL, et al. Kidney disease characteristics, prevalence, and risk factors in León, Nicaragua: a population-based study. BMC Nephrology. 2023;24(1). doi: 10.1186/s12882-023-03381-1.

27. Gonzalez-Quiroz M, Heggeseth B, Camacho A, Oomatia A, Al-Rashed AM, Zhang Y, et al. Population-level detection of early loss of kidney function: 7-year follow-up of a young adult cohort at risk of Mesoamerican nephropathy (vol 53, dyad151, 2023). International Journal of Epidemiology. 2024;53(1):1. doi: 10.1093/ije/dyad163. PubMed PMID: WOS:001185398100001.
